# Beeinträchtigung der Blutversorgung durch Cerclagen: Mythos oder Realität? Ein Überblick über die experimentelle Studienlage

**DOI:** 10.1007/s00113-020-00847-x

**Published:** 2020-08-19

**Authors:** Stefan Förch, Sabrina Sandriesser, Annabel Fenwick, Edgar Mayr

**Affiliations:** 1 Klinik für Unfallchirurgie, Orthopädie, plastische und Handchirurgie, Universitätsklinikum Augsburg, Stenglinstr. 2, 86156 Augsburg, Deutschland; 2 Institut für Biomechanik, Berufsgenossenschaftliche Unfallklinik Murnau, Murnau, Deutschland

**Keywords:** Reposition, Frakturversorgung, Knochenbruchheilung, Pseudarthrose, Biologische Knochenheilung, Reduction, Operative treatment of fractures, Fracture healing, Pseudarthrosis, Biological bone healing

## Abstract

**Einleitung:**

Die Verwendung von Cerclagen zur Osteosynthese ist ein kontrovers diskutiertes Thema. Als Gegenargument gelten negative Effekte auf die periostale Blutzirkulation. In der vorliegenden Arbeit soll die vorhandene Evidenz geprüft werden, ob Cerclagen tatsächlich zu einer relevanten Reduktion der periostalen Durchblutung führen.

**Methodik:**

In einer systematischen Literaturrecherche wurde nach experimentellen Studien gesucht, die sich mit dem Einfluss von Cerclagen auf die periostale Blutversorgung befassen.

**Ergebnis:**

Es existiert keine experimentelle Studie, die die Auswirkung von Cerclagen auf die Durchblutung von frakturierten Knochen untersucht. Es konnten 7 experimentelle Arbeiten identifiziert werden. Hierunter befanden sich 2 Arbeiten an menschlichen Kadaver-Femora, die keine relevante Reduktion der Blutversorgung zeigten. Die übrigen 5 Untersuchungen wurden im Tiermodell an lebenden Versuchstieren durchgeführt. Hier wies eine Studie an Kaninchen-Femora szintigraphisch eine postoperativ um 45–56 % reduzierte Perfusion nach. Eine Studie an Pferde-Radii sowie drei Studien an Hunde Femura ergaben hingegen mikroangiographisch keine relevante Einschränkung der Blutversorgung. Eine Studie wurde hierbei am osteotomierten, die anderen an unverletzten Knochen durchgeführt.

**Diskussion:**

Im Tiermodell konnte lediglich in einer Studie direkt postoperativ eine relevante Reduktion der Blutversorgung durch Cerclagen belegt werden. In 4 anderen Untersuchungen am Tiermodell über längere postoperative Zeiträume zeigte sich die Durchblutung der Knochen hingegen nicht beeinträchtigt. Auch in 2 Versuchsreihen an menschlichen Kadaver-Femora ließen sich keine relevanten negativen Effekte nachweisen.

Zumindest in mittel- bis langfristigem Verlauf lässt sich somit die Befürchtung einer relevanten Beeinträchtigung der Blutversorgung durch Cerclagen für unverletzte oder osteotomierte Knochen nicht durch experimentelle Studien belegen. Zu frakturierten Knochen existieren keine experimentellen Studien.

## Abstract (en)

**Introduction:**

The use of a cerclage for osteosynthesis is a controversially discussed topic. They are said to damage the periosteal blood circulation and therefore impair bony healing. This article examines the available evidence on whether cerclages actually lead to a relevant reduction in periosteal perfusion.

**Methods:**

A systematic review of the literature was performed in the PubMed library, searching for experimental studies concerning the influence of cerclages on periosteal blood supply.

**Results:**

No experimental study exists which used a fracture model to investigate the influence of cerclages on the periosteal blood supply of fractured bones. A total of seven experimental studies could be identified. Of these studies two used human cadaver femora, which showed no relevant impairment of the blood supply. The other five investigations were animal experiments carried out on live animals. In rabbit femora the blood perfusion was shown by scintigraphy to be postoperatively decreased by 45–56% . In contrast, three other studies using dog femora and one using equine radii showed no relevant impairment of the periosteal blood supply. One study used an osteotomy model, the others used intact bones.

**Conclusion:**

Only one study using a rabbit model could demonstrate a relevant reduction of the periosteal blood supply by cerclages. In four other investigations on animal models over longer postoperative time periods the blood perfusion of the bones showed no impairment. In two series of experiments on human cadaveric femora no negative effects were also found. At least in the mid-term and long-term run the fear that cerclages could impair the blood supply of intact bone or postosteotomy cannot be confirmed by experimental studies. There is no experimental study using a fractured bone model.

## Einleitung

Die Verwendung von Cerclagen in der Knochenbruchbehandlung ist ein kontrovers und teilweise auch emotional diskutiertes Thema. Als Vorteile werden eine erhöhte Stabilität und eine bessere Reposition angeführt. Für subtrochantere Frakturen konnte dies auch in Studien belegt [4, 7, 10] und z. T. sogar eine schnellere Heilung gezeigt werden [2].

Als Gegenargument gegen die Verwendung von Cerclagen gilt die Befürchtung, durch die zirkuläre Kompression die periostale Blutzirkulation zu kompromittieren und dadurch die Frakturheilung zu stören [11].

In der vorliegenden Arbeit soll daher überprüft werden, ob diese Befürchtung durch experimentelle Untersuchungen belegt werden kann.

## Methodik

In einer systematischen Literaturrecherche wurde in der *PubMed*-Datenbank unter den Stichwörtern „cerclage“, „blood supply“ und „blood circulation“ nach experimentellen Studien gesucht, die den Einfluss von Cerclagen auf die periostale Durchblutung untersuchen. Die Ergebnisse wurden von E.M. und S.F. unabhängig anhand der Abstracts gescreent. Ausgeschlossen wurden alle nichtexperimentellen Arbeiten (Fallserie, klinische Studien, Reviews, Leserbriefe etc.). Eingeschlossen wurden ausschließlich experimentelle Arbeiten, die sich mit dem Effekt von Cerclagen auf die periostale Blutversorgung befassten. Deren Volltexte und Quellenangaben flossen in die weitere Analyse ein.

## Ergebnisse

**Figure 1:**
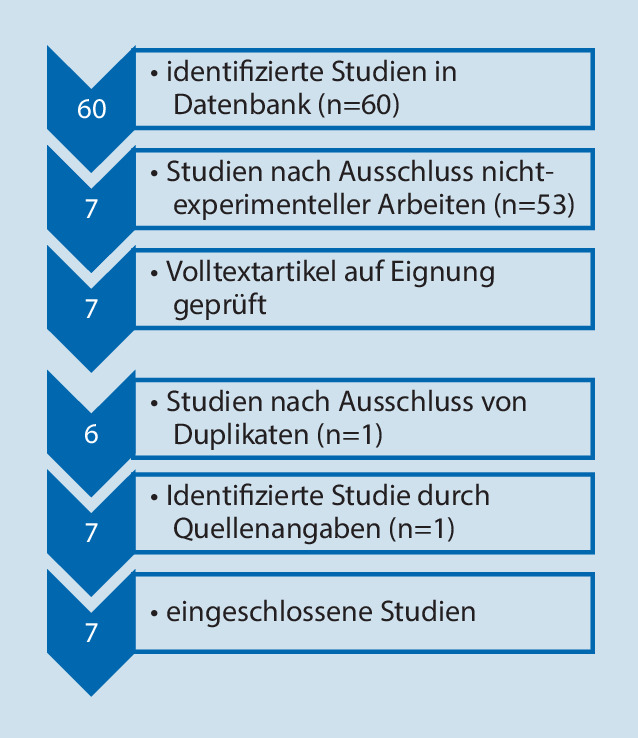

Unter den genannten Suchbegriffen fanden sich 60 Artikel. Anhand der Analyse der Abstracts zeigten sich 53 als nichtexperimentelle Arbeiten und wurden entsprechend ausgeschlossen. 7 experimentelle Studien konnten in eine Volltextanalyse eingeschlossen werden. Hierbei stellten sich 2 Arbeiten [12, 13] als Doppelpublikation heraus. Über die Quellenangaben konnte eine weitere experimentelle Studie identifiziert werden [8]. Es konnten somit 7 experimentelle Studien zur Knochendurchblutung nach Cerclagen-Anlage identifiziert werden (Abb. 1).

**Figure 2:**
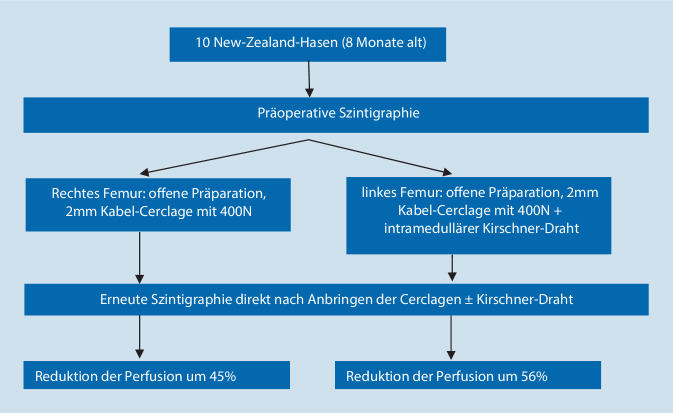

Die aktuellste Studie stammt aus dem Jahr 2016 von Karakoyun et al. [3]. Der schematische Studienablauf ist in Abb. 2 wiedergegeben. Die Implantation der Cerclagen an den unverletzten Kaninchen-Femora erfolgte offen über 1 cm lange Inzisionen. Szintigraphisch wurde prä- und postoperativ in Früh- und Spätphase die arterielle Durchblutung gemessen. Als „region of interest“ wurde ein Areal von 2 × 1 cm um die Cerclage gewählt. Postoperativ zeigte sich eine Abnahme der Perfusionsrate um 45 % (*p* = 0,001) für die rechte (ohne intramedullären Draht) und um 56 % (*p* < 0,001) für die linke (mit intramedullärem Draht) Seite. Die Autoren stellten somit eine signifikante Reduktion der Durchblutung durch die Cerclagen fest.

Drei Studien wurden an Hunde-Femora durchgeführt [5, 9, 12, 13].

**Figure 3:**
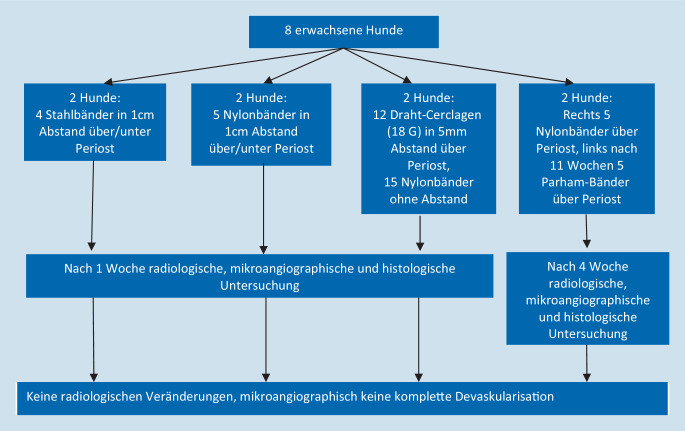

Kirby und Wilson [5] brachten bei insgesamt 8 erwachsenen Hunden Stahl- bzw. Nylonbänder und Stahl-Cerclagen in unterschiedlichen Dicken und Konfigurationen an unverletzten Femur-Schäften an (Abb. 3). Die Stahlbänder und Cerclagen wurden hierbei nach Gefühl angespannt, bis sie nicht mehr longitudinal am Knochen bewegt werden konnten. Gerade bei Draht-Cerclagen entspricht dies der gängigen chirurgischen Praxis. Die Nylonbänder hingegen wurden nach Herstellerangaben mit einer entsprechender „Applikationspistole“ je nach Größe mit 80, 178, 222, 534 bzw. 778 N angespannt und angebracht. Hier wurden also z. T. deutlich höhere Verschlusskräfte aufgewendet als bei „Cable“-Cerclagen für humane Zwecke üblich.

6 Hunde wurden eine Woche postoperativ, 2 Hunde nach 4 Wochen eingeschläfert, eine Gefäßperfusion durchgeführt und anschließend die Femora fixiert und präpariert. Die Proben wurden dann in 2 Ebenen geröntgt und mikroangiographisch auf das Vorhandensein von gefüllten kortikalen Gefäßen untersucht. Histologisch wurde nach Hämatoxylin-Eosin-Färbung der Anteil der gefüllten bzw. leeren Lakunen bestimmt.

In keiner Auswertung zeigten sich Unterschiede zwischen den verschiedenen Cerclage-Typen oder den verschiedenen angelegten Spannungen der Nylon-Cerclagen, wobei diese sich zum Zeitpunkt der Präparation alle gelockert zeigten. Diese Lockerung wurde auf Wasserabsorption zurückgeführt.

Radiologisch bestanden keine auffällige Knochenreaktion, periostale Proliferation oder kortikale Erosion. In allen Versuchsanordnungen konnten zahlreiche gefüllte Blutgefäße direkt unter und zwischen den Cerclagen nachgewiesen werden. Trotz der zahlreichen und teilweise ohne Abstand voneinander eingebrachten Cerclagen zeigte sich in keinem Fall eine komplette kortikale Devaskularisation.

Histologisch zeigten sich in den Kontroll-Femora 24 ± 3 % leere Lakunen, in den operierten Femora 28 ± 17 %. In der periostalen Schicht sowohl unter als auch zwischen den Cerclagen zeigten sich signifikant mehr leere Lakunen als in den kortikalen oder endostalen Schichten bzw. als in periostalen Schichten nichtoperierter Femora. Da sich keine Unterschiede zwischen den Regionen unter bzw. zwischen den Cerclagen ergaben, wurde dies als Auswirkung der Präparation und nicht der Cerclagen selbst gewertet. Da auch bei nichtoperierten Femora leere Lakunen zu finden waren, schlussfolgern die Autoren, dass diese auch der Präparation geschuldet oder ein Normalbefund sein könnten. Eine Korrelation mit den mikrovaskulären Untersuchungen ergab sich nicht. Ein Rückschluss auf die Durchblutung oder Vitalität des Knochens könne durch das Fehlen von Osteozyten in den Lakunen allein nicht erfolgen.

Insgesamt kommen die Autoren zum Schluss, dass die Vaskularisierung durch Cerclagen und Bänder bis 10 mm Breite nicht beeinträchtigt wird.

**Figure 4:**
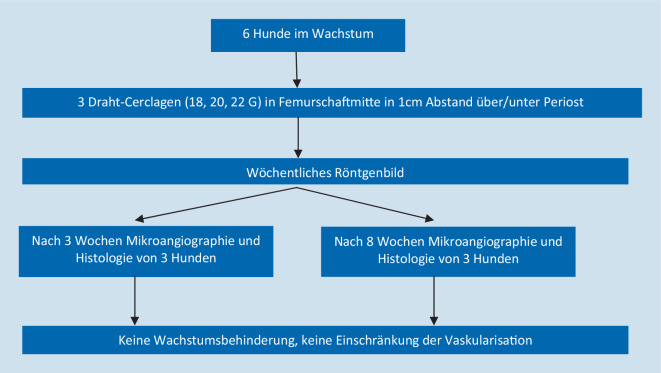

Eine weitere Studie von Wilson [12, 13] befasst sich mit den Auswirkungen von Cerclagen auf Femora von noch im Wachstum befindlichen Hunden. Den Studienablauf gibt Abb. 4 wieder. Alle Cerclagen wurden mithilfe eines speziellen Spanners (Rhinelander Wire Thightner-Twister, Richards Manufacturing Company, Inc., Memphis, TN, USA) mit derselben Spannung angelegt; die genaue Spannung wird nicht angegeben.

Bei einer Platzierung der Cerclagen über dem Periost zeigten sich keine radiologischen Auffälligkeiten oder anatomische Veränderungen. Bei einer Platzierung unter dem Periost kam es 2 Wochen nach Einbringen der Cerclagen zu periostalen Knochenappositionen im Bereich der Linea aspera. Diese kondensierten nach 5 und verschwanden nach 8 Wochen. In keiner Gruppe war die Vaskularisierung mikroangiographisch beeinträchtigt; vor allem nach 3 Wochen zeigte sich eine fast schon überschießende Reaktion mit zahlreichen kortikalen und periostalen Gefäßen auch unter den Cerclagen. Auch nach 8 Wochen traten keine Nekrosen auf; an zahlreichen Stellen waren die Cerclagen von neu gebildetem Knochen eingemauert. Unterschiede zwischen den verschiedenen Drahtstärken bestanden nicht.

Im Gegensatz zur Studie von Kirby et al. [5] konnten histologisch keine leeren Lakunen nachgewiesen werden. Es konnte weder eine Osteonekrose noch das Vorhandensein von Entzündungszellen nachgewiesen werden.

Die Autoren schlussfolgern, dass Cerclagen den im Wachstum befindlichen Knochen nicht devitalisieren und das Knochenwachstum nicht behindern.

**Figure 5:**
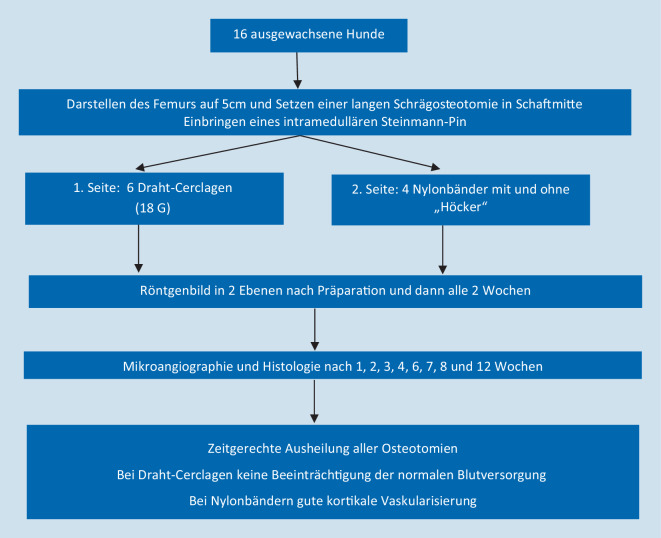

Rhinelander et al. [9] untersuchten als einzige osteotomierte und damit nicht mehr intakte Knochen (Abb. 5). Die Draht-Cerclagen wurden wie bei Wilson et al. [12] mithilfe eines speziellen Instrumentariums mit gleicher Spannung angebracht, die auch hier nicht exakt genannt wird. Die Nylonbänder wurden ebenfalls über das entsprechende Instrumentarium mit definierter Spannung angebracht. Ein Teil der Nylonbänder besaß auf der Innenseite „Höcker“, die ähnlich dem „Limited-contact“-Prinzip von Plattenosteosynthesen eine geringere Auflagefläche haben und damit eine geringere Kompromittierung der Blutversorgung gewährleisten sollten. Histologisch und radiologisch zeigten alle Osteotomien jeweils eine zeitgerechte knöcherne Konsolidierung. Mikroangiographisch ergab sich insbesondere für die Draht-Cerclagen keine Störung der normalen kortikalen Blutversorgung oder kortikalen Knochenheilung. Ein Unterschied zwischen den Bändern mit oder ohne Höcker bestand nicht.

**Figure 6:**
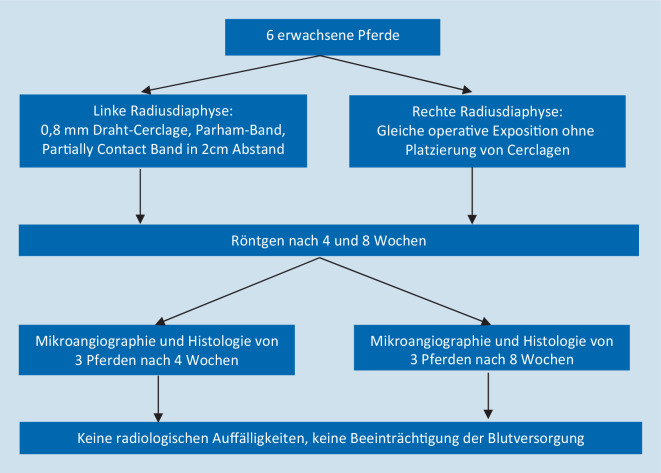

Eine Studie [8] existiert zu equinen Radii (Abb. 6). Die Anspannung der Cerclagen erfolgte jeweils über spezielle Applikationsgeräte mit definierter, aber nicht näher genannter Spannung. In den durchgeführten Röntgenaufnahmen zeigten sich keine Auffälligkeiten. Zu keinem Zeitpunkt bestand/bestanden histologisch eine erhöhte Porosität des Knochens, osteozytenfreie Lakunen oder eine erhöhte Osteoklastenaktivität, unabhängig vom Cerclagen-Typ. Sowohl in der Cerclagen- als auch in der Kontrollgruppe konnten zahlreiche endostale und wenige periostale Blutgefäße nachgewiesen werden. Ein Unterschied zwischen den Gruppen bzw. den verschiedenen Cerclage-Typen oder eine Beeinträchtigung der Blutversorgung durch die Cerclagen bestand nicht.

Nyrop et al. [8] kommen somit zum Schluss, dass korrekt platzierte Cerclagen die kortikale Durchblutung nicht obstruieren, merken allerdings an, dass diese bei Pferden stärker ausgeprägt ist als z. B. bei Kaninchen oder Menschen.

**Figure 7:**
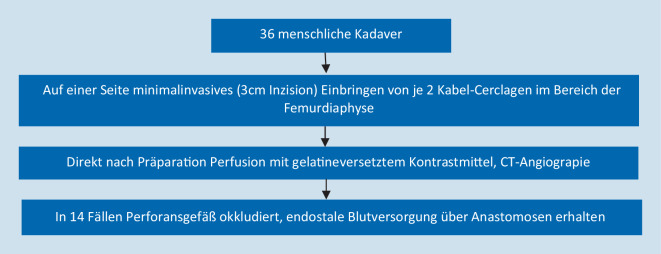

Zu Cerclagen an menschlichen Knochen konnten 2 Studien identifiziert werden:

Apivatthakakul et al. [1] verwendeten 18 menschliche Kadaver. Der Studienablauf wird in Abb. 7 skizziert. Es wurde jeweils eine Seite präpariert und die Cerclagen mit einem speziellen minimal-invasiven Instrumentarium eingebracht; eine langstreckige Präparation oder Freilegung des Femurs wurde nicht durchgeführt. Die Cerclagen wurde mit einem Drahtspanner mit gleicher, aber nicht genau genannter Spannung angezogen. Die Gegenseite wurde nicht präpariert und diente als Vergleichsgruppe.

In 11 Fällen wurde ein, in 3 Fällen 2 Perforansgefäße durch die Cerclagen okkludiert. Die Durchblutung wurde jedoch in allen Fällen über Anastomosen sichergestellt, eine Beeinträchtigung der endostalen Blutversorgung ergab sich nicht. Einschränkend ist anzumerken, dass keine Mikroangiographie durchgeführt wurde. Zudem sind die Ergebnisse nicht direkt auf eine Fraktursituation oder ausgedehnte chirurgische Präparation mit dadurch geschädigter Vaskularisation übertragbar.

**Figure 8:**
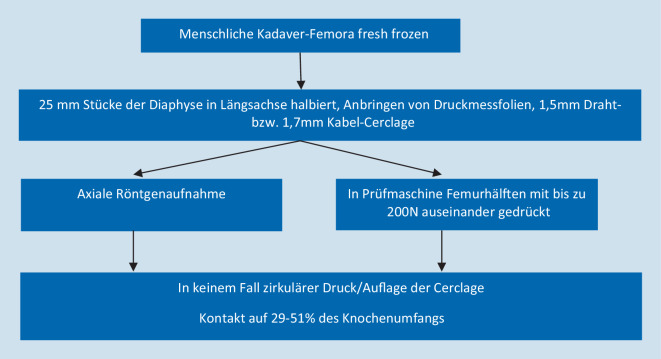

Eine andere Studie an menschlichen Kadaver-Femora [6] untersuchte die Kontaktfläche und Druckverteilung von Draht- und Kabel-Cerclagen an der Knochenoberfläche (Abb. 8). Beide Cerclagen-Typen zeigten sowohl in den Röntgenaufnahmen als auch bei der Druckmessung nur punktuellen und keinen zirkulären Knochenkontakt. In Abhängigkeit von der Knochengeometrie übten Draht-Cerclagen auf 29–39 % der gesamten Zirkumferenz Druck aus, Kabel-Cerclagen auf 31–52 %. Zu einem kompletten zirkulären Kontakt mit daraus resultierender Strangulation kam es in keinem Fall. Diese Werte gelten allerdings nur für die untersuchte Femur-Diaphyse, da sie extrem von der jeweiligen Knochengeometrie abhängig sind.

## Diskussion

Insgesamt gibt es in der Literatur nur wenige Studien, die sich experimentell mit der Auswirkung von Cerclagen auf die regionale Durchblutung des Knochens beschäftigen; keine davon nutzt ein Frakturmodell. Überwiegend wurden die Studien an Hunde-Femora durchgeführt [5, 9, 12, 13]. Hier ergeben sich natürlich Einschränkungen hinsichtlich der Übertragbarkeit auf menschliche Knochen mit z. T. geringerer Durchblutung und dünnerem Periost [8]. Vergleichbare experimentelle Studien an lebenden Menschen verbieten sich natürlich, sodass letztendlich dennoch auf Tiermodelle zurückgegriffen werden muss.

Des Weiteren ist zu hinterfragen, inwieweit eine Post-mortem-Angiographie den In-vivo-Zustand widerspiegelt. Bei Apivatthakakul et al. [1] erfolgte diese bei konservierten humanen Kadavern und ist somit nicht als physiologisch anzusehen. Bei den meisten anderen verwendeten Präparationsmethoden [5, 8, 9, 12] hingegen wurde die Perfusion direkt nach Töten der Tiere mit konstantem Druck von 120 mm Hg durchgeführt. Dies entspricht nicht ganz der physiologischen, biphasischen Perfusion. Es wurde jedoch versucht, diese so gut als möglich zu simulieren. Es erscheint bei dieser Präparation zumindest unwahrscheinlich, dass bei der Post-mortem-Perfusion mehr Blutgefäße perfundiert und dargestellt werden als in vivo.

Bei der Interpretation der Studien muss als weitere Einschränkung genannt werden, dass keine Frakturmodelle verwendet wurden und somit Aussagen zur Durchblutung in der Fraktursituation nicht möglich sind. Ein chirurgisches Zugangstrauma erfolgte jedoch in jedem Fall. Kirby et al. [5] begründen den Verzicht auf ein Frakturmodell damit, dass sie nur die isolierten Effekte von Bändern auf die Blutversorgung untersuchen und die Auswirkungen einer möglicherweise unzureichenden Stabilisierung einer Fraktur durch die Bänder ausschließen wollten. Karakoyun et al. [3] befürchteten einen Einfluss eines Frakturhämatoms auf die szintigraphische Messung. Apivatthakakul et al. [1] geben an, eine Veränderung der Gefäßanatomie durch eine Fraktur vermeiden zu wollen. Nyrop et al. [8] weisen darauf hin, dass durch den begleitenden Weichteilschaden die Durchblutungsverhältnisse beeinflusst werden könnten und dies in zukünftigen Studien beachtet werden sollte. Nur eine einzige Arbeit untersuchte osteotomierte und damit nicht mehr intakte Knochen [9]. Auch die Osteotomie lässt sich hinsichtlich des Weichteilschadens und damit ggf. zusätzlicher Kompromittierung der Durchblutung nicht 1:1 auf eine Fraktursituation übertragen. Nach durchgeführter Osteotomie kann zumindest eine Aussage über die Auswirkung der Cerclage auf die Knochenheilung erfolgen, hier konnten keine negativen Effekte nachgewiesen werden.

Die Art, Applikation, Menge und Positionierung der Cerclagen variieren in den einzelnen Untersuchungen.

In den Studien, die verschiedene Cerclage-Typen (Stahl- oder Draht-Cerclagen, Stahl- oder Nylonbänder) verglichen, konnten zum Großteil [5, 9] keine Unterschiede zwischen den verschiedenen Modellen gefunden werden. Lenz et al. [6] konnten hingegen zeigen, dass Draht-Cerclagen eine signifikant höhere Auflagefläche auf dem Knochen haben als Stahl-Cerclagen. Verschiedene Breiten [5] von Bändern bis 10 mm schienen keinen Einfluss auf die Blutzirkulation zu haben.

Die Cerclagen wurden teilweise nach Gefühl [5], teilweise mit definierter Spannung über spezielle Applikationsgeräte [8, 9, 12] angezogen. Im operativen Alltag werden Draht-Cerclagen oft ebenfalls nach Gefühl, Cable-Cerclagen in der Regel mit speziellen „Spannern“ mit maximal 50 N (z. B. Cable Cerclagen ø 1,7 mm; Fa. DePuy Synthes Companies, Oberdorf, Schweiz, „product number“ 298.801.01) angebracht. Hinsichtlich der Spannung ist die Präparation in den Studien also bei Draht-Cerclagen dem operativen Vorgehen vergleichbar. Hier konnte in keinem Fall eine relevante Beeinträchtigung der Blutversorgung nachgewiesen werden. Kirby und Wilson [5] verwendeten u. a. Nylonbänder mit steigender Spannung von 80 bis 778 N. Diese zeigten sich bei der Präparation allerdings alle gelockert; ein Zusammenhang zwischen Anspannung und Durchblutung oder gar ein „Cut-off“-Wert konnte nicht ermittelt werden.

**Figure 9:**
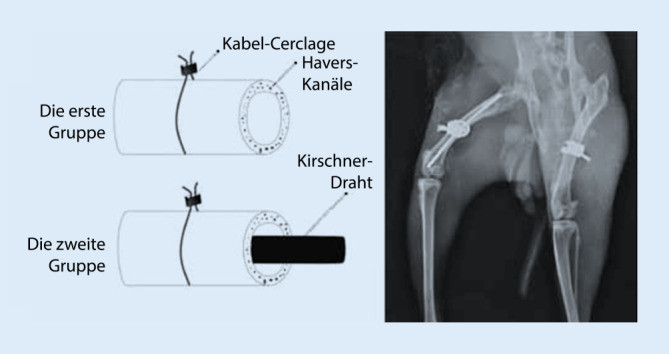

Bei Draht-Cerclagen zeigen sich hingegen erhebliche Unterschiede der eingesetzten Spannung zum gängigen operativen Vorgehen. In der einzigen Studie [3], die tatsächlich eine relevante Reduktion der Blutzufuhr nachweisen konnte, wurden Draht-Cerclagen verwendet und mit 400 N gespannt. Im Gegensatz zu den anderen Tierstudien handelt es sich hier um einen recht kleinen Knochen (Kaninchen; Abb. 9); die eingebrachten Cerclagen sind im Verhältnis zudem extrem groß. Gängige, an menschlichem Knochen angewandte Cerclage-Systeme (z. B. Cable Cerclagen ø 1,7 mm; Fa. DePuy Synthes Companies, Oberdorf, Schweiz, Product number 298.801.01) werden hingegen nur mit maximal 50 N angespannt. Warum so hohe Druckwerte gewählt wurden, wird nicht erläutert, nur darauf hingewiesen, dass der Druck pro Fläche unabhängig von der Größe des Knochens und der Cerclage ist. Ob die hohen Anspannungskräfte tatsächlich oder allein der Grund für die kompromittierte Durchblutung sind, lässt sich anhand der Studienlage nicht beantworten. Auch Messmethode und Zeitpunkt könnten hier eine Rolle spielen. Die Durchblutung wurde von Karakoyun et al. [3] nicht mikroangiographisch, sondern szintigraphisch und direkt postoperativ bestimmt. Verlaufskontrollen nach Tagen oder Wochen wie in den anderen Studien wurden nicht durchgeführt. Möglicherweise kommt es bei dem sehr frühen Zeitpunkt durch das operative Trauma zu einer passager messbaren Reduktion der Perfusionsrate, die sich im weiteren Verlauf wieder normalisiert. Auch Interspeziesunterschiede könnten für die differenten Ergebnisse eine Rolle spielen [8].

Eine maximale oder optimale Kraft, mit der Cerclagen angespannt werden sollten, lässt sich aus den untersuchten Studien nicht ableiten.

Auch bezüglich der eingesetzten Menge der Cerclagen zeigten sich große Variationen zwischen einzelnen Cerclagen im Abstand von mehreren Zentimetern bis hin zu ohne Abstand gelegten Bändern über eine Strecke von 3 cm. Bezüglich einer Kompromittierung der Blutversorgung konnten dennoch keine Unterschiede aufgezeigt werden.

In allen Studien wurden die Cerclagen diaphysär platziert. Inwieweit sich metaphysäre Cerclagen bezüglich der Durchblutung anders verhalten, lässt sich somit nicht ableiten. Aus der theoretischen Überlegung, dass der metaphysäre Kochen prinzipiell besser vaskularisiert ist, könnte man mutmaßen, dass die Auswirkung einer Cerclage hier sogar noch geringer ausfällt.

**Figure 10:**
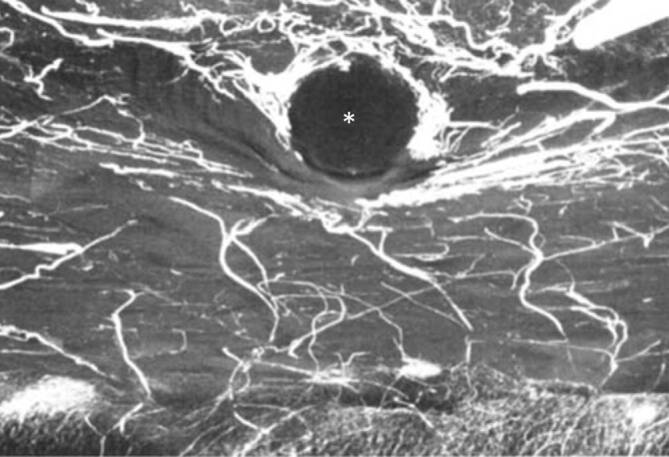

Es lässt sich zusammenfassen, dass sich in keiner Studie an Hunden eine relevante Kompromittierung der Blutzufuhr zeigte. In der Regel konnten mikroangiographisch Blutgefäße auch im Bereich direkt unter den Cerclagen zahlreich nachgewiesen werden (exemplarisch Abb. 10). Radiologisch bestand keine Atrophie oder Pseudarthrosenbildung. Auch auf im Wachstum befindlichen Knochen [8, 12, 13] wurde kein negativer Einfluss nachgewiesen. Für Pferde-Radii zeigten sich vergleichbare Ergebnisse, auch wenn die Autoren anmerken, dass die Blutversorgung beim Pferd stärker ausgeprägt ist als bei Kaninchen oder Menschen.

**Figure 11:**
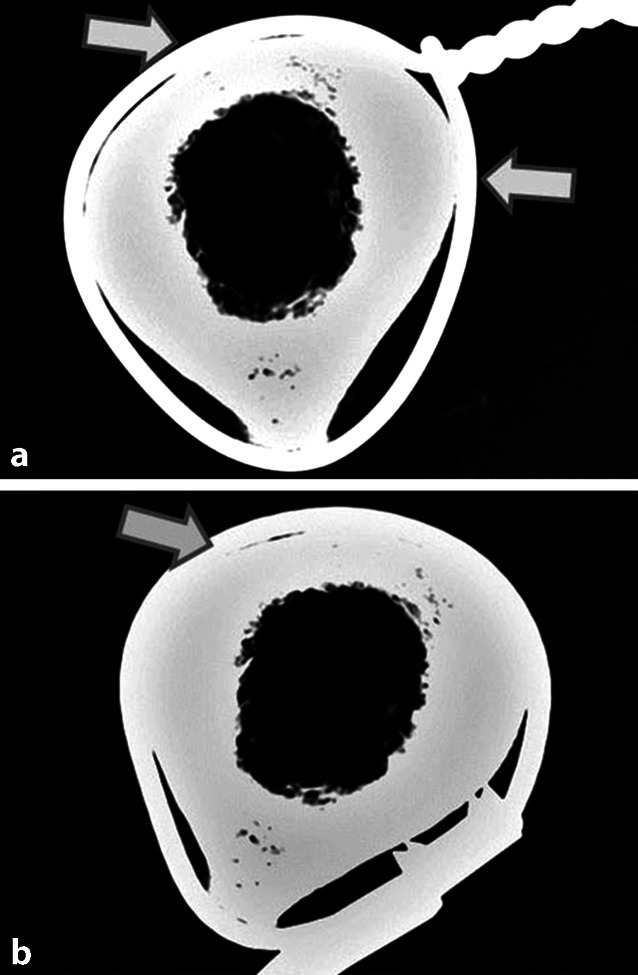

Zwei Studien existieren zu menschlichen Kadaver-Femora [1, 6]. Hier konnte zum einen [1] angiographisch gezeigt werden, dass beim minimal-invasiven Einbringen durchaus Makrogefäße gefährdet sind und durch die Cerclage okkludiert werden können. Dank guter Kollateralisierung hatte dies jedoch keine Auswirkung auf die Mikrozirkulation. Zum anderen wurde nachgewiesen [6], dass Cerclagen an der Femurdiaphyse nie komplett zirkulär, sondern nur punktuell am Knochen anliegen und damit keine Strangulation mit Unterbrechung der kompletten Blutzufuhr entstehen kann (Abb. 11).

Zusammenfassend lässt sich somit sagen, dass keine experimentelle Studie existiert, die die Auswirkung von Cerclagen am frakturierten Knochen untersucht. Die Frage, wie sich Cerclagen in einer Fraktursituation auf die periostale Durchblutung auswirken, kann daher nicht beantwortet werden. Alle vorhandenen Untersuchungen wurden an intakten oder osteotomierten Knochen durchgeführt. An diesen konnte eine Reduktion der Knochendurchblutung nur in einer einzigen Studie direkt postoperativ nachgewiesen werden. Die Mehrzahl der Studien zeigt in dem für die Knochenheilung relevanten Zeitintervall keine Kompromittierung der Blutversorgung und keine Beeinträchtigung des Knochenwachstums oder der Knochenheilung nach Osteotomie. Inwieweit sich die Ergebnisse der Tierversuche auf den Menschen übertragen lassen, ist unklar, aber auch in den Untersuchungen an menschlichen Kadaverknochen wurde keine substanzielle Beeinträchtigung der Durchblutung durch Cerclagen nachgewiesen.

Anhand der vorhandenen experimentellen Studien zur Auswirkung von Cerclagen auf die periostale Blutversorgung kann die Befürchtung einer Strangulation und eine Störung der Knochenheilung zumindest am intakten oder osteotomierten diaphysären Knochen nicht untermauert werden.

## Fazit für die Praxis

Aufgrund fehlender experimenteller Arbeiten bleibt die Auswirkung von Cerclagen auf die periostale Blutversorgung in der Fraktursituation unklar; hier sind weitere experimentelle und klinische Studien erforderlich.

Die vorhandene experimentelle Studienlage rechtfertigt weder eine generelle Ablehnung noch den unkritischen klinischen Einsatz von Cerclagen. Wenn sie zur Reposition oder Erhöhung der Stabilität erforderlich sind, ist auf eine entsprechend schonende Operationstechnik und eine möglichst geringe Anzahl zu achten.
